# Negative pressure pulmonary edema: a case report

**DOI:** 10.1186/s12871-019-0730-x

**Published:** 2019-05-01

**Authors:** Jun Xiong, Yongxing Sun

**Affiliations:** Department of Anesthesiology, Sanbo Brain Hospital, Capital Medical University, No. 50, Yikesong, Xiangshan, Haidian District, Beijing, 100093 China

**Keywords:** Negative pressure pulmonary edema, Hypocortisolism, Hypothyroidism, Obstructive sleep apnea

## Abstract

**Background:**

The negative pressure pulmonary edema is rare clinical situation which caused mainly by upper airway obstruction. However except upper airway obstruction, there may be other pathophysiological disorders making patients more vulnerable to pulmonary edema. Based on these disorders, upper airway obstruction is the trigger to induce negative pressure pulmonary edema.

**Case presentation:**

This case was a 5-year-old girl with tumor on saddle area, her hormones level were abnormal preoperatively, such as cortisol, adrenocorticotrophic hormone, free T4 and total T4. During the stage of induction, negative pressure pulmonary edema took place due to mild upper airway obstruction. And the instant chest Computer tomography proved diagnosis clue. After intensive care, most lung field of this girl recovered to normal within 48 h.

**Conclusion:**

The patient with abnormal hormone levels is vulnerable to pulmonary edema, mild upper airway obstruction triggered negative pressure pulmonary. Thus pre-operation hormones supplement is as important as keeping upper airway unobstructed.

## Background

Negative pressure pulmonary edema (NPPE) is an uncommon and life threatening complication of general anesthesia. Its incidence is 0.1% of general anesthesia with tracheal intubation, mostly caused by laryngospasm [1]. In other words, although patients may breathe laboriously due to upper airway obstruction, they rarely develop NPPE in clinical [2]. We report a case of instant pulmonary edema following elective general anesthesia using sevoflurane inhalation induction, who did not experience obvious laryngospasm peri-induction, but with hypothyroidism and hypocortisolism, We explore the possibility of pulmonary edema was exactly unapparent negative pressure generation, but also ask whether the other agents should be considered as culprits.

## Case presentation

**Fig. 1:**
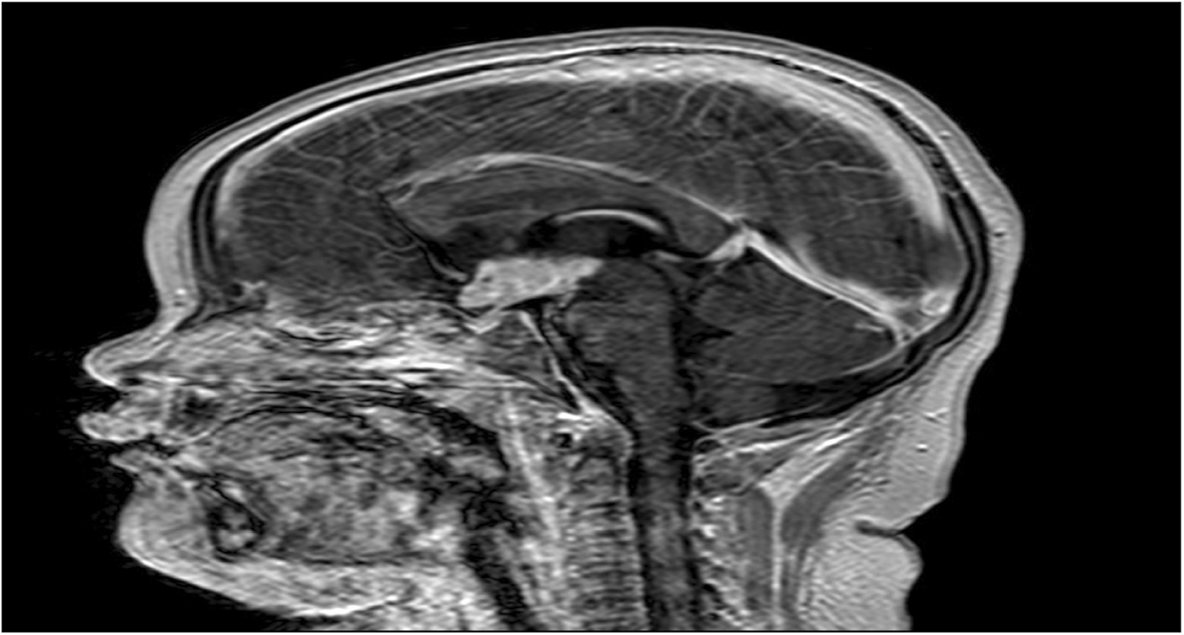
Tumor on saddle area. The tumor is T1 hypointensity, T2 hyperintensity and homogenous enhancement

A 5-year-old female child was admitted for intracranial tumor on 29th March 2018. Her chief complaint was polydipsia and polyuria for 3 years. The MRI image of December 2015 in local hospital showed tumor on sellar region, with characteristics of T1 hypointensity, T2 hyperintensity and homogenous enhancement. Recent 2 months, she presented with symptoms of intermittent body seizure and unconsciousness. The reexamination MRI in March 2018 revealed the tumor was bigger (Fig. 1). Her height was 95 cm and weight 32 kg, the body mass index was 35.5. Her previous history was negative. Physical examination indicated no obvious signs except obesity and short neck. Although without results of polysomnography test, apnea did take place during sleep, so we inferred there was possibility of obstructive sleep apnea (OSA). We evaluated her with ASA II and Mallampati III. Results of blood routine, coagulation function and D-dimer tests were normal. But the blood electrolytic, such as serum Na^+^ (154.8 mmol/L) and Cl^−^ (119 mmol/L) were significantly higher than the reference. Meanwhile some blood hormone results were also abnormal. For example, free T4 (0.56 ng/ml) and total T4 (3.27μg/ml) were slightly lower than the reference, but cortisol (1.41μg/dl) and adrenocorticotropin (< 5.0 pg/ml) were significantly reduced. These changes of hormone demonstrated pituitary dysfunction and might cause the electrolytic and distribution of body fluids abnormal. Electrocardiogram and echocardiogram tests were normal, but serious fatty liver was detected by abdominal ultrasound. Thus, for exact diagnosis and treatment in progress, the operation with general anesthesia would be done to draw the tumor tissue for pathological examination.

**Fig. 2:**
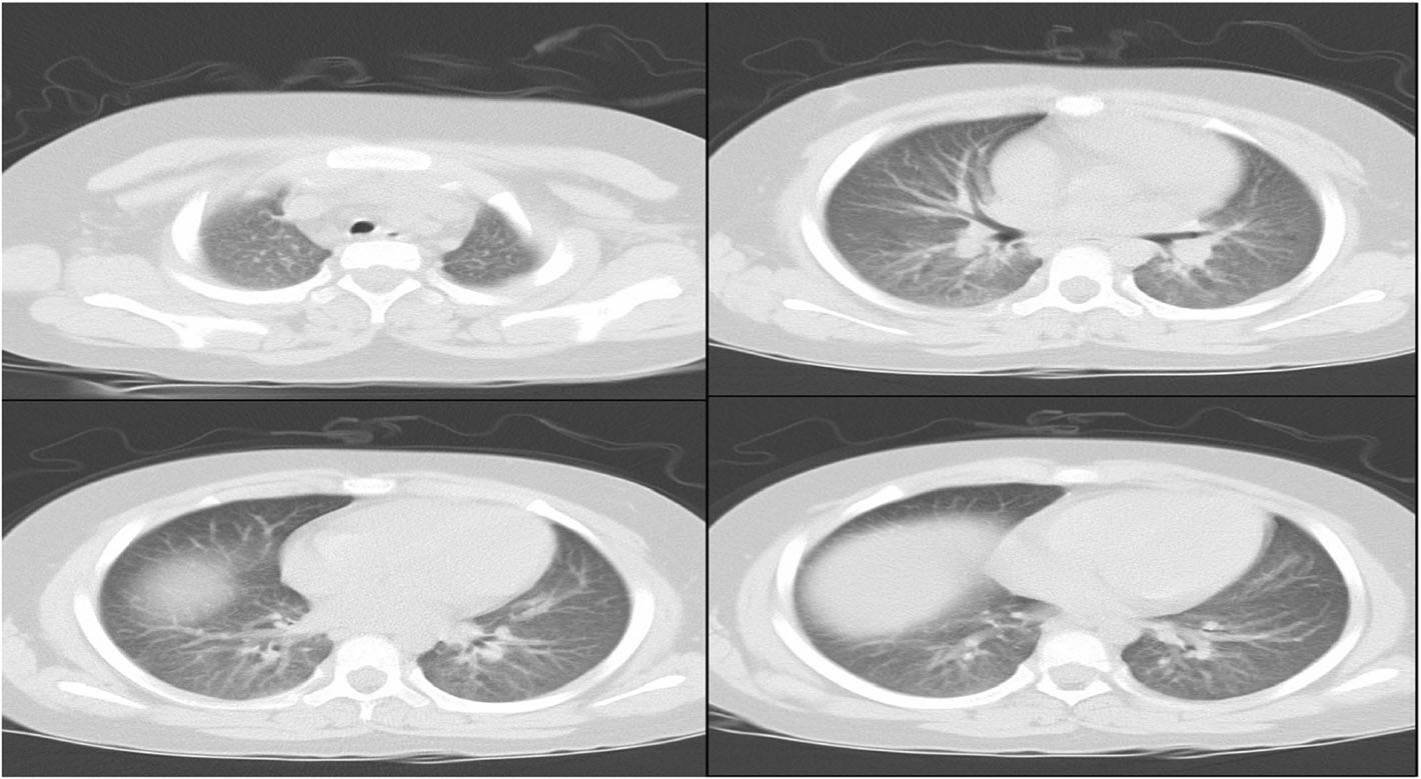
Chest CT of 30th March (Before the operation). This CT examination showed all lung fields were clean

After admission, sodium valproate 0.5 g/bid and levothyroxine 25μg/qn were administered. Blood electrolytic was monitored and regulated daily. The result of chest computer tomography (CT) on 30th March was negative, and her preoperative chest examination was normal (Fig. 2). Na^+^ 146.6 mmol/L and Cl^−^ 111.6 mmol/L, which re-examined on 3rd April, closed to normal range. Routine cortisol supplement was delayed due to results of cortisol and adrenocorticotropin reported not in time.

On 4th April, this girl was sent to the operating-room by stretcher. It was difficult to establish venous access because she was obese and incompatible, so the sevoflurane inhalation induction was given with 3%~ 8% sevoflurane and O_2_ 6L/min. At the beginning of induction, she struggled for few seconds. The monitor showed SpO_2_ was 92%~ 96%. Although she was keeping spontaneous breathe, the tidal volume was very low, just 70~100 ml. Meanwhile, her breathe movement was not as same as normal, because there was very mild collapse of suprasternal fossa on inhalation stage, albeit it was not apparent. At the same time, another anesthesiologist was called for more assistance because of difficult jaw-thrust. By squeezing breather bag, support ventilation following her spontaneous breathe was tried, but the situation could not be improved and jaw-thrust was still difficult.

Twenty minutes later, venous access was established. Thus propofol 50 mg, sulfentanil 15μg and rocuronium 40 mg were administered. Endotracheal intubation with 4.5-intensive-tube was successful with routine laryngoscope, because exposing and visualizing her larynx and glottis was easily. No secretion was found in her mouth. And her tonsilla and vocal cords were normal. During the induction phase, although her heart rate and blood pressure waved normally, low tidal volume, low SpO_2_ and mild airway blocking sustained, the SpO_2_ was only 91%~ 95% with FiO_2_ 100%.

**Fig. 3:**
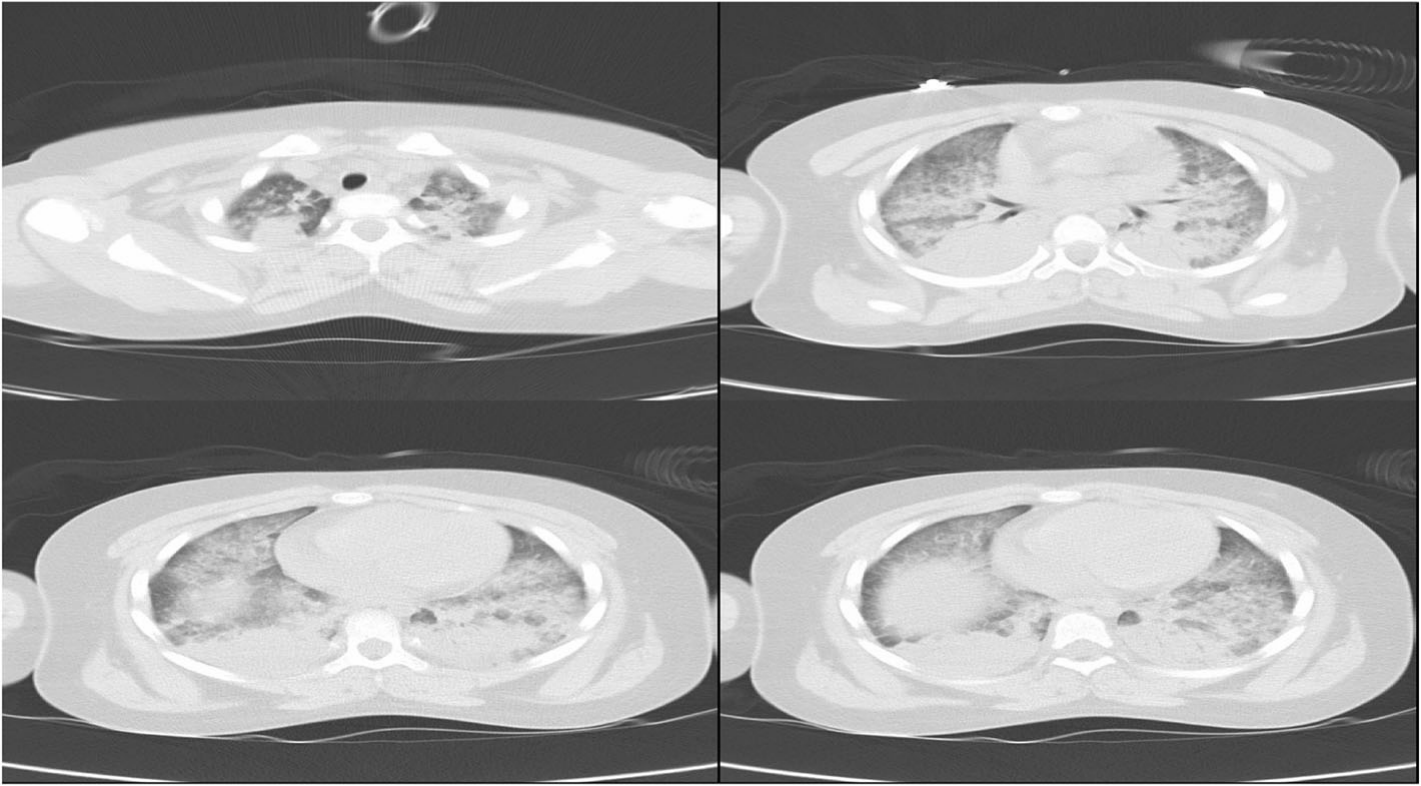
Chest CT of 4th April (The day of operation). These images demonstrated bilateral pulmonary consolidation accompanied by air bronchogram and the ground-glass opacity lesions

After endotracheal intubation, mechanical ventilation setting was as follows: tidal volume 200 ml, I:E = 1:2, f 20, FiO_2_ 100%. The monitor showed seriously high peak pressure sustaining at 36~39cmH_2_O. In the meanwhile the SpO_2_ was only 90%~ 92% and EtCO2 more than 55 mmHg. Although no wheezing rale and stridor, obvious blistering sound and coarse crackles presented in both lung fields by chest auscultation. Thus airway was aspirated immediately, only little secretion in the tracheal. But the ventilation was not ameliorated. After changing volume controlling model into pressure controlling model, ventilation situation was still bad. Then general anesthesia was enhanced by increasing concentration of sevoflurane and administering sulfentanil 10μg and rocuronim 20 mg. At the same time, methylprednisolone 40 mg and furosemide 10 mg were administered. Unfortunately, ventilation situation was not improved, such as seriously high airway peak pressure and low SpO_2_. Then the operation had to be cancelled, and she was sent to ICU after taking chest CT scan (Fig. 3). Surprisingly, chest CT showed bilateral pulmonary consolidation accompanied by air bronchogram and the ground-glass opacity lesions, but echocardiography demonstrated a preserved left ventricular ejection fraction 65%.

**Fig. 4:**
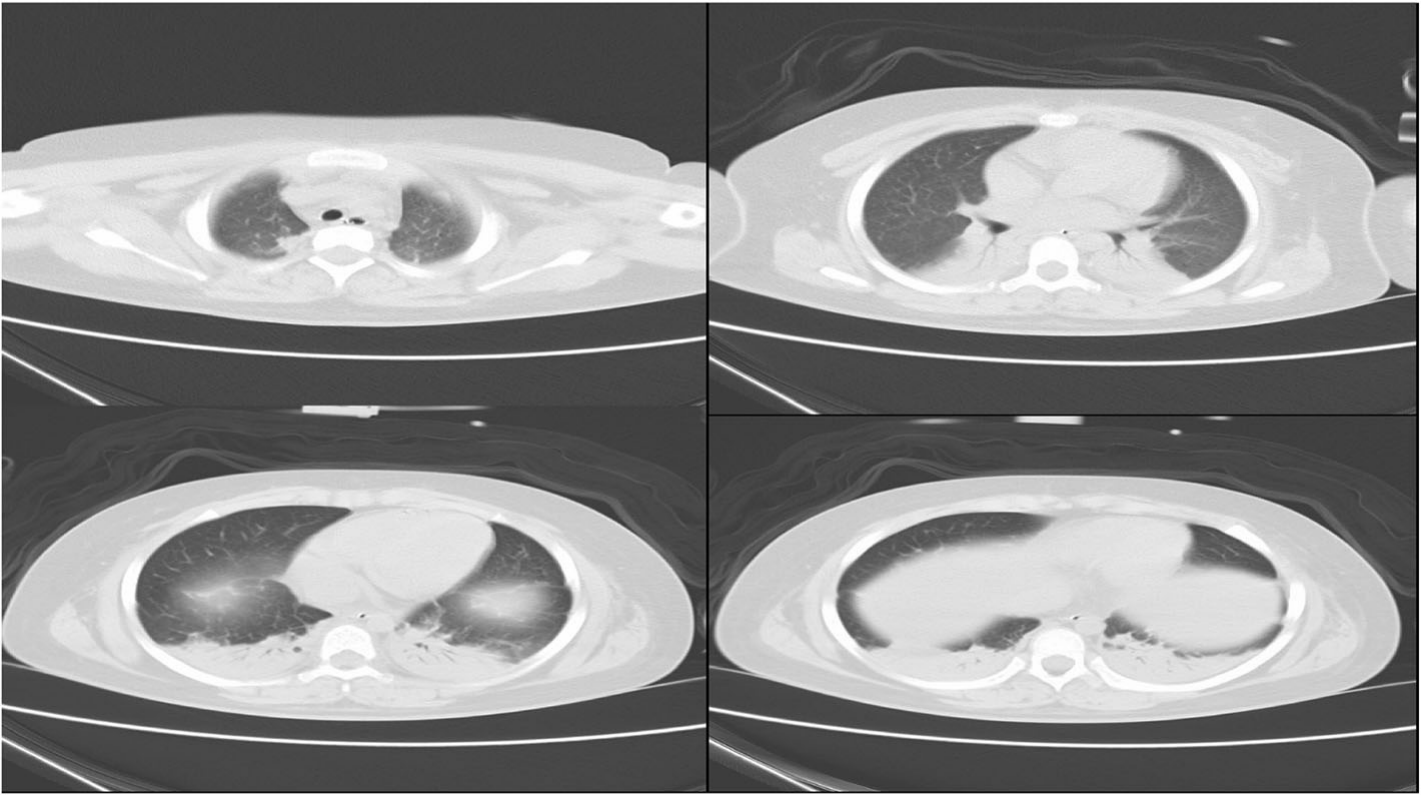
Chest CT of 6th April (after 48 h of ICU supportive care). This revealed the most of lung field was normal except the bilateral lower lobe alveolar infiltrates and consolidation

In ICU, positive pressure mechanical ventilation was initiated. Methylprednisolone 40 mg and levothyroxine 25μg for one dose were administered. Although there was no bacterial organisms in sputum culture, meropenem was administered for anti-infection. Several hours later, breathe sound was obviously better except the bottoms of bilateral lung with tiny rale. After 48 h of supportive care in ICU, the pulmonary edema resolved rapidly, reexamined chest CT demonstrated the most of lung field was clean except bilateral lower lobe alveolar infiltrates and consolidation (Fig. 4).

## Discussion and conclusion

NPPE, also known as postobstructive pulmonary edema, develops in patients with spontaneous respiratory effort who have upper airway obstruction and generate very negative intrathoracic pressure leading to sever hypoxemia and pulmonary edema [3].

Most children cases of NPPE have been caused by glottis or subglottic obstruction [4]. But causes of adult NPPE are not as same as the children’s. The most common reported reason for adult experiencing NPPE is post-extubation laryngospasm [5], even the incidence of NPPE is more than 50% among men following laryngospasm [6]. In this case, although there were no apparent causes discussed above, mild collapse of suprasternal fossa and difficult jaw-thrust during induction phases predicted the possibility of upper airway obstruction. In clinical practice, when unexplained pulmonary edema takes place, NPPE should be considered in different diagnosis, although it is uncommon [3]. In the meanwhile, this case included other similar aspects of NPPE, for example, rapid onset of pulmonary edema after efforts at inspiration against obstructive airway [3] and rapid resolves within 12 to 24 h [7]. In addition, normal ejection fraction also led us from cardiogenic pulmonary edema to non-cardiogenic pulmonary edema.

In this case, there were other agents to prompt the development of NPPE, such as abnormal hormones and possible OSA. Because sellar region tumor disturbed function of pituitary endocrine, so the serum levels of free T4, total T4, cortisol and adrenocorticotropin were abnormal. Her serum cortisol and adrenocorticotropin were significantly lower than normal level, which have been well known as stress hormone. And its abnormity is a signal of dysfunction of hippocampus-pituitary-adrenal axis. Patients with hypocortisolism may be more vulnerable to lung leaking syndrome. Vice verse, pulmonary edema may be a symptom of adrenal insufficiency. Therefore glucocorticoids have been widely used to treat this syndrome. Clinically, hydrocortisone effectively reduces tracheal aspirate fluid volume and oxygen dependency. And hydrocortisone may improve capillary permeability as well as lung inflammatory reaction [8]. For this girl, because results of cortisol and adrenocorticotropin were not reported in time, she was not treated with cortisol preoperation. Thus she confronted with the danger developing pulmonary edema before operation. The treatment of cortisol supplement was started in operating-room, following more active cortisol supplement in ICU. Improved serum cortisol level protected alveolar and vessel endothelial intact [9] to improve pulmonary edema.

Except of cortisol reduction, her free T4 and total T4 were lower than the reference range, which was hypothyroidism. It has been well known that edema is the most obvious sign of patients with hypothyroidism. If hypothyroidism was severe, cardiogenic pulmonary edema would happen because of the loss of inotropic and chronotropic effects of thyroid hormone [10]. Even non-cardiogenic pulmonary edema could be caused indirectly because hypothyroidism results in leakage of plasma protein and increases capillary permeability [11]. Additionally, hypothyroidism is considered as one potential cause of upper airway obstruction [12]. A variety of factors may be involved, such as alteration in ventilator drive, obesity, and so on [13]. If a patient was with hypothyroidism complication with obesity and OSA, there is more probability to develop non-cardiogenic pulmonary edema [14]. This girl’s weight was 32 kg and BMI was greater than 35, she was not only obese, but also hypothyroidism. And she was with possibility of OSA, so she was coincidence with above situation and vulnerable to non-cardiogenic pulmonary edema.

Before operation, the results of her chest CT on 30th March was negative, which reported by radiology department. However we thought the images of CT showed slight effusion on both lung fields, or more blood in pulmonary circulation. When compared with images of CT on 6th April, the performance of effusion was more obvious on former CT (all of images of chest CT can be obtained from the corresponding author). In fact, this girl’s hormone disorders were able to produce pulmonary edema CT images, there was no contradiction between CT images and her illness. But different photographic condition might cause this deviation of interpretation of chest CT. Whatever results of chest CT, preoperative hormone disorder were putting her to the marginal of pulmonary edema.

In the present case, this girl was obese. And physical examination showed short neck and obvious apnea during sleep. Although there was lack of polysomnograph test, she was great possibility of OSA. If there is unexplained postoperative pulmonary edema in patients without laryngospasm history, OSA should be considered as one of culprits [3, 15]. A trait of OSA is frequent episodes of intermittent hypoxia, which leads to pulmonary vascular dysfunction by damaging vascular endothelial cells [16]. Based on pulmonary vascular dysfunction, this girl was susceptible to leaky lung syndrome. Because destructive vascular endothelial increases permeability of lung capillaries and leads more fluids into pulmonary interstitium. This might be the reason of increased oxygen and pressure dependency. In fact, these signs predicted severe lung leaking syndrome.

The girl was evaluated with Mallamti III preoperatively. And physical examination demonstrated short neck, obese and apnea during sleep. During inhalation induction jaw-thrust was difficult. All these factors indicated the possibility of upper airway obstruction. Mild collapse of suprasternal fossa was another evidence of obstructive upper airway. However there was lack of laryngospasm evidence, because of no signs of larynospasm, such as wheezing rale and stridor. When intubation, no secretion was found on her cords and her glottis opened normally. So larynospasm was not the prior consideration. Although no larynospasm, we suspected mild upper airway obstruction was able to trigger NPPE easily because she possessed too much risks of pulmonary edema.

In ICU, positive pressure ventilation, diuretics and other treatment were administered. Positive pressure ventilation alleviated negative airway pressure in chest. Diuretics were used for the aim of conservative fluid strategy. Hormone supplementary therapy protected alveolar and vessel endothelial intact. Hormones improve pulmonary edema by cAMP-indepentent mechanisms, such as cortisol and thyroid hormone. This girl was not only lack of cortisol, but also free T4 and total T4. So combined administration of them were able to resolve NPPE quickly [17]. Although she recovered as soon as the most cases of NPPE, she had been confronted with severe hypoxemia due to massive pulmonary edema and shunt. After 5 days of actively supportive care, she was discharged from the ICU.

In current case, although there was no apparent laryngospasm, on base of mild upper airway obstruction, all other causes, for example hypocortisolism, hypothyroidism, and OSA, worked tighter to trigger and accelerate NPPE. So we speculated the pathophysiology of this case was that, the tumor on sellar region damaged pituitary endocrine function to produce hormones disorder. This abnormity promoted body fluid distribution disorder and increased pulmonary capillary permeability. In other words, the tumor made the girl vulnerable to pulmonary edema. At last, mild upper airway obstruction triggered the cascade of NPPE.

In this case, there was the biggest regret that the girl’s family members rejected second opportunity of operation. But for us, there were some apocalypses. Firstly, we should open mind to look for a pathology that would explain the whole clinical scenario. Secondly, supplement of hormones should be sufficient to sustain electronic at normal range before operation. Lastly, the process of anesthesia induction, supraglottic airway device should be used to keep airway unobstructed. The normal ventilation may be the most important to avoid NPPE.

## Abbreviations

CT: Computer tomography
NPPE: Negative pressure pulmonary edema
OSA: Obstructive sleep apnea
